# Towards a basis for vascular versus intestinal phenotype differences; East Meets West in Behcet’s disease

**DOI:** 10.3389/fimmu.2026.1758222

**Published:** 2026-07-17

**Authors:** Kerem Abacar, Tom Macleod, Fatma Alibaz-Oner, Haner Direskeneli, Dennis McGonagle

**Affiliations:** 1 Leeds Institute of Rheumatic and Musculoskeletal Medicine, University of Leeds, Chapel Allerton Hospital, Leeds, United Kingdom; 2 Department of Internal Medicine, Division of Rheumatology, School of Medicine, Marmara University, Istanbul, Türkiye; 3 National Institute for Health and Care Research (NIHR) Leeds Biomedical Research Centre, Leeds Teaching Hospitals NHS Trust, Leeds, United Kingdom

**Keywords:** Behçet’s disease, inflammatory bowel disease, intestinal development, MEFV, vasculitis

## Abstract

Phenotypic differences in disease expression across ethnic populations may hold the key to a better understanding of the pathophysiology of Behçet’s disease (BD), which has a wide geographical distribution and marked clinical variability between East Asian, Middle Eastern, and Western countries. In contrast to the predominance of vascular involvement in regions such as Turkey, intestinal involvement is more frequently observed in East Asian patients, particularly in Japan. Genome-wide association studies in inflammatory bowel disease (IBD), which shows clinical similarity to BD, have identified NOD2 and IL23R variants as major susceptibility loci in European populations. However, the relative absence of these IBD-related variants in East Asian populations, where intestinal BD is more prevalent, suggests the presence of alternative, BD-specific mechanisms driving gut involvement in different genetic backgrounds. Herein, we argue that this diversity in BD clinical expression may also reflect underlying differences in innate immune responses, particularly involving IL-1 signaling pathways. The striking prominence of *MEFV* variants (approximately 20% carrier frequency) in Turkish populations where vascular BD is more common, compared with their relative scarcity in East Asia, may offer important clues as to how population-specific genetic backgrounds shape distinct inflammatory phenotypes. In this article, we discuss these immunogenetic contrasts in relation to clinical heterogeneity, with a particular focus on the IL-1 pathway regulated by MEFV, to provide a more nuanced understanding of ethnic differences in BD pathogenesis and their implications for future therapeutic strategies.

## Introduction

Behçet’s disease (BD) is a complex inflammatory condition mediated by autoimmune and innate immune-activated mechanisms responsible for a wide spectrum of serious organ involvements beyond oral-genital aphthous ulcers, including ocular, central nervous system, and vascular and intestinal inflammation (1). Although inflammatory diseases exhibit some heterogeneity across global populations, one of the most interesting BD features is a more extreme ethnic phenotypic diversity. The biggest phenotypic difference between Turkey, where its prevalence is highest in the world, and the Far Eastern populations is the dichotomy between vascular and intestinal BD distribution. While intestinal BD is more common in Asia, it is rare in Turkey, where conversely vascular BD is much more common (2).

While ethnic differences exist in other autoimmune diseases between the East and West, such as multiple sclerosis, these can be explained by different patterns of autoantibody associations (3). By contrast, the striking disparities in Behçet’s disease (BD) remain largely unexplained. In this paper, we propose one potential, though not exclusive, mechanism that may contribute to the divergent distribution of major BD phenotypes across populations, focusing on the influence of *MEFV*-regulated IL-1 biology. We link this IL-1 framework to inflammatory bowel disease (IBD), where the reported prevalences of ulcerative colitis (UC) and Crohn’s disease (CD) are 12.53/10^5^ and 31.83/10^5^, respectively, in Turkey (4) and 133.2/10^5^ and 31.9/10^5^ in Japan (5). The disproportionately high prevalence of UC, rather than CD, in Japan is a key observation because intestinal BD in East Asia often presents with a UC-like pattern. This UC-skewed epidemiology strongly implicates mucosal barrier dysfunction, IL-10–dependent regulatory pathways, and population-specific microbiome signatures as central factors shaping gut-predominant BD in East Asian cohorts. Coupled with the established role of IL-1 in immunothrombosis, these contrasts form the basis of a model that may explain why intestinal BD predominates in Japan, whereas vascular BD is more common in Turkey.

## Prevalence of major vascular BD and intestinal BD

The prevalence of vascular BD varies between 5% and 30% (6). There are studies reporting the frequency of major vascular involvement in BD as 25%, 27.7%, and 31.1% in Turkey, the country with the highest prevalence, but in Japan, it is only 6% (7–10). In a multicenter study from Turkey conducted by Alibaz-Oner et al., 84.6% of vascular BD (n = 220) patients had only venous disease, 8.1% (n = 21) had only arterial disease, and 4.2% (n = 11) had both venous and arterial diseases. Furthermore, deep vein thrombosis (70.5%) in the lower extremity was the most common major vascular involvement (9).

In contrast, the frequency of gastrointestinal involvement is only 2.8% in Turkey, 4% in Saudi Arabia, 10% in China, 32% in Taiwan, and 50%–60% in Japan, as the country with the highest prevalence of intestinal BD (11), which may mirror the higher background rate of UC in Japan, as already mentioned. Additionally, this distinction appears between intestinal and vascular BD in cluster studies in the same ethnic population. In a cohort-study in China, BD clustered into five phenotypic categories. The percentages of vascular and intestinal involvement in the clusters were as follows: cluster 1: vascular: 0%, intestinal: 0%; cluster 2: vascular: 0%, intestinal: 14.5%; cluster 3: vascular: 0%, intestinal: 92.3%; cluster 4: vascular: 0%, intestinal: 5.6%; cluster 5: vascular + cardiac: 44.3% + 33.6%, intestinal: 6.1% (12). The pathogenetic scenarios underpinning intestinal and vascular BD discordance is thus far poorly understood.

## HLA-B*51 and other MHC associations—East Meets West

The strongest genetic factors for BD implicate the MHC-I-opathy spectrum disorders. These factors include HLA-B*51 genetic risk loci are summarized in **Table 1**, *endoplasmic reticulum amino-peptidase 1 (ERAP1), IL23R, MICA, IL12, IL10, IFNGR1*, and *STAT4* which collectively drive tissue-specific immune perturbations and aberrant CD8 T-cell responses across different ethnic populations (13). Among these factors, the potential impact of HLA-B*51 on the disease phenotype is non-negligible. The largest meta-analysis showed that HLA-B*51 carriage in BD is associated with an increased prevalence of genital ulcers, ocular and skin manifestations, and a decreased prevalence of gastrointestinal involvement, while neither increasing nor decreasing vascular manifestations (14). Given its lack of association with intestinal BD, HLA-B*51 is unlikely to explain the East–West variation in disease phenotype.

**Table 1:** Certain single nucleotide polymorphisms that were shown to be associated with Behçet's Disease.

Gene	SNP
*HLA*	HLA-B*51, HLA-A*26, HLA-A*31, HLA-C*0704
*ERAP1*	rs17482078, rs10050860, rs13154629, rs1065407, rs2287987, rs2013717,
*IL10*	rs1518111, rs1800872, rs1800871, rs1495965, rs2222202, rs1518110, rs3021094
*IL10RA*	rs2228054, rs2228055
*IL23R-IL12RB2*	rs924080, rs17375018, rs7517847, rs10489629, rs1343151, rs1495965, rs1495966, rs12141431, rs4655535, rs6665569, rs11209032, rs34426521, rs12119179
*STAT4*	rs7574865, rs7574070, rs7572482, rs897200
*IL23R*	rs17375018, rs7517847, rs1343151
*MICA*	rs3094584
*STAT3*	rs2293152
*TNFAIP3*	rs9494885, rs10499194, rs7753873
*CCR1-CCR3*	rs7616215
*IL12*	rs17810546, rs1874886
*KLRC4*	rs2617170
*MEFV*	rs61752717
*TRAF5*	rs12569232, rs6540679
*TRAFIP2*	rs13210247
*IL12A-AS1*	rs17810546
*PSORS1C1*	rs12525170
*IL18RAP*	rs2058660
*NOD1*	rs2075818
*IL1A-IL1B*	rs3783550
*IL1RL-IL18R1*	rs12987977, rs12999364, rs4851569
*IL27*	rs153109
*TNFα*	rs1800629
*IFGNR1*	rs9376268, rs4896243
*IL35*	rs428253

HLA-B*51 may not be a key discriminator between different Turkish and Asian phenotypes. The prevalence of HLA-B*51 is 13.6%-24% (15, 16) in the healthy population in Turkey and 13.8%-22% in healthy Japanese populations (15, 17). However, the frequency of HLA-B*51 in BD is 70%-75% (15, 16) in Turkey and 57.9% in Japan (15). In the phenotypic cluster analysis of BD in Japan, the cluster (cluster 2) with the lowest rate of HLA-B*51 positivity (33%) had the highest frequency of gastrointestinal (75.7%) involvement. In addition, this cluster had the lowest rate of pathergy positivity (31.8%) and the lowest rate of fulfilling the International Study Group (ISG) classification criteria (76.4%) (18). In one of the largest BD cohort studies in Japan (n=3044), gastrointestinal involvement was less common in HLA-B*51-positive BD patients compared to HLA-B*51-negative patients (22.9% vs. 31.8%). However, the 22.9% frequency in the HLA-B*51-positive group remains significantly higher than the gastrointestinal BD frequency reported in Turkey (19). It should also be noted that HLA-B51 positivity is accompanied by more cutaneous findings.

Importantly, differences in the classification criteria used across countries may partly account for this apparent discrepancy. The International Study Group (ISG) criteria, more frequently used in Turkey and Europe, do not include gastrointestinal involvement, whereas the Japanese criteria explicitly recognize it as a specific disease subtype (20). This distinction may therefore contribute to the higher reported prevalence of gastrointestinal BD in Japanese cohorts, although the inclusion of gastrointestinal features in the Japanese criteria itself may have been driven by the historically greater frequency of this phenotype in that population. Relative to other phenotypes, the diminished reliance of intestinal involvement on MHC-I underscores the amplified significance of alternative genetic risk factors. Moreover, the lower frequency of pathergy and HLA-B51 positivity in intestinal BD may contribute to diagnostic overlap with IBD, especially in patients who exhibit extraintestinal manifestations (19).

Beyond studies that exclusively focused on HLA-B*51, the relationship between HLA-A*26 (another gene linked to BD) and HLA-B*51 and clinical manifestations have been investigated in another Japanese cohort. HLA-A*26 was detected more frequently in patients with co-occurring ocular and gastrointestinal involvement (HLA-A*26 positive vs. negative: n = 5/21, 23.8% vs. n=0/58, 0%, respectively; p=.001). Although the trends suggest a possible interaction between HLA-A*26 and certain BD phenotypes, the low number of cases in each subgroup (e.g., only 5 HLA-A*26-positive patients with ocular and GI involvement) limits the strength of these observations. Neither HLA-A*26 nor HLA-B*51 was associated with an increased frequency of gastrointestinal involvement alone. The frequency was 42.9% in HLA-A*26-positive patients compared to 36.2% in HLA-A*26-negative patients (n = 9 vs. n = 21; p = 0.61). Similarly, the frequency was 29.8% in HLA-B*51-positive patients compared to 40.8% in HLA-B*51-negative patients (n = 14 vs. n = 20; p = 0.29) (21). Also, the frequency of intestinal BD does not appear to be increased in HLA-B*51-negative BD patients in Turkey (16).

This “differential immunopathology” in BD whereby HLA-B*51 is not linked to all BD manifestations is also shared with other MHC-1-opathies where, for example, HLA-B*27 is not linked to intestinal inflammation in ankylosing spondylitis (22). This points to genes outside the MHC, environmental factors, or both as being key for gut involvement. Beyond genetic predisposition, substantial differences in environmental exposures between Japanese and Turkish populations, including dietary composition, microbiota structure, and hygiene-related factors, may critically influence mucosal immune programming. In particular, long-term dietary habits rich in fermented and fiber-based foods in East Asia have been linked to distinct gut microbial signatures and short-chain fatty acid profiles, whereas westernized dietary patterns are associated with pro-inflammatory dysbiosis (23–25). Such environmental pressures may drive epigenetic modifications in immune-regulatory genes, shaping the organ tropism and inflammatory phenotype of BD.

Taken together, both genetic and non-genetic influences appear to interact in determining disease expression across populations. All these data highlight that HLA-B*51 may be one of the causes of phenotypic differences between ethnic populations. However, the effect of HLA-B*51 alone appears to be relatively modest in a highly complex disease such as BD, indicating the need for factors beyond HLA-B*51. Recently, *SLCO4A1* single-nucleotide polymorphism (SNP) has been identified in association with BD ocular involvement (rs6062789: OR = 0.41 [95% CI = 0.30-0.58], p-value = 1.92 × 10-7) and *DDX60L* SNP with neurological involvement (rs62334264: OR = 4.12 [95% CI 2.34 to 7.24], p-value = 8.85 × 10-7) (26). However, these SNPs are shared across global populations and would not explain the disparity between vascular and intestinal BD.

## NOD2 and IL23R polymorphisms in IBD: East vs. West

**Figure 1:**
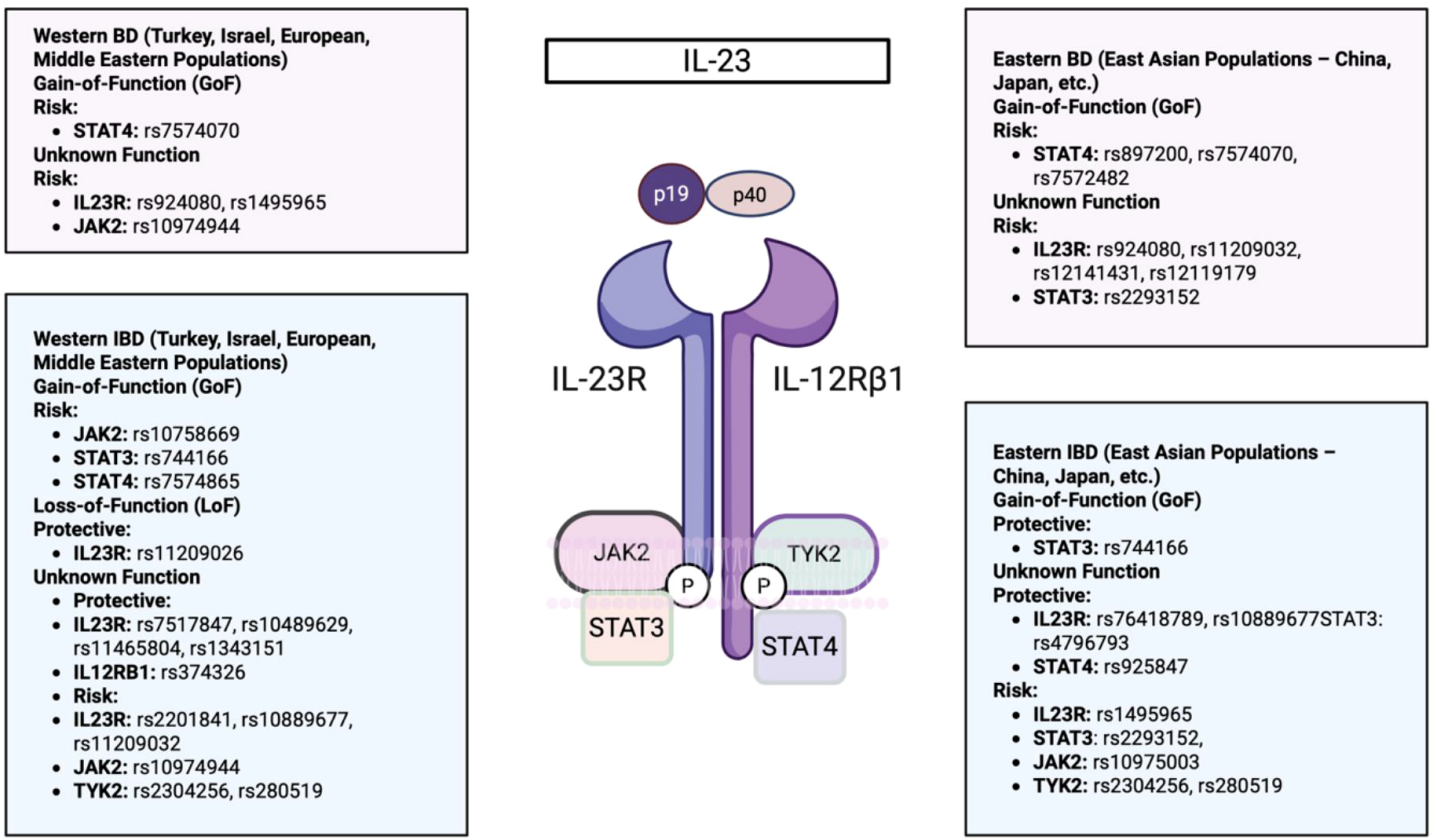
Population-specific functional SNPs in the IL-23/IL-12 and JAK-STAT pathways in IBD and Behçet’s disease. Single nucleotide polymorphisms (SNPs) in IL23R, IL12B, TYK2, JAK2, STAT3, and STAT4 are functionally implicated in inflammatory bowel disease (IBD) and Behçet’s disease, with population-specific patterns. European cohorts predominantly exhibit loss-of-function variants in IL23R and TYK2, and gain-of-function or regulatory variants in IL12B, STAT3, and JAK2. In East Asian populations, IL23R and STAT3 gain-of-function variants are more prominent in both IBD and Behçet’s disease, while protective variants affecting TYK2 and IL23R signaling have also been identified.

The two major cytokines involved in intestinal inflammation, including IBD and BD enteritis, are IL-23 and IL-10, as demonstrated by GWAS studies (27, 28). A meta-analysis comparing European and non-European populations revealed a high degree of genetic correlation in IBD susceptibility (Crohn’s disease rG = 0.76; ulcerative colitis rG = 0.79) (29), suggesting shared underlying mechanisms. While European risk variants in genes such as *NOD2* are largely absent or significantly less frequent in East Asian populations, alternative *IL23R* risk variants have been identified in both populations, highlighting distinct population-specific variant profiles despite shared pathway involvement (29–31). *IL23R* is a globally relevant gene for intestinal inflammation, with both East Asian and European populations showing risk-associated SNPs. However, the specific risk variants and their population frequencies differ significantly, suggesting that although IL-23 pathway involvement is shared, the genetic architecture is population-specific (**Figure 1**).

In European populations, *NOD2* mutations impair bacterial sensing and autophagy, triggering chronic inflammation. These risk variants, however, are rare or absent in East Asian populations, suggesting alternative inflammatory pathways. Indeed, *TNFSF15–TNFSF8* and *ATG16L1* polymorphisms, which exhibit significant heterogeneity, may be more relevant in this population. *IL23R* remains a globally relevant gene in intestinal inflammation; however, the specific risk variants and their effect sizes differ substantially across populations. While well-established *IL23R* SNPs such as rs11209026 confer protection in European cohorts, they are largely absent in East Asians. Instead, East Asian studies identify alternative *IL23R*-linked SNPs, including rs76418789 and rs1495965, which map to the same locus but represent population-specific variants with distinct effect sizes and functional relevance. Moreover, several additional components of the IL-23/Th17 axis, including *TYK2*, *STAT3*, and *IL12B*, harbor functional variants that differ in frequency and impact across populations (**Figure 1**). These findings emphasize that although the IL-23 signaling pathway remains central, its genetic architecture is markedly population-specific (29, 32, 33).

Although population-specific *IL23R* variants have been identified, their functional significance remains unclear. This pathway shows only weak links to thrombotic inflammation, being primarily associated with mucosal immunity and gut-predominant disease, supported by murine models whereby IL-23 minicircle induces arthritis rather than thrombosis.

## IL-10: a gatekeeper of intestinal homeostasis and its divergent roles in IBD and BD

Although the IL-23 and IL-10 signaling pathways are central to IBD pathogenesis across populations, the specific genetic variants contributing to these pathways differ considerably between regions. For example, *IL23R* risk variants vary between East Asian and European cohorts, as outlined above. In contrast, IL-10 remains a consistently important regulator of intestinal homeostasis across populations, modulating immune responses and preserving epithelial barrier integrity. How these cytokine pathways intersect with BD pathogenesis, particularly in the setting of intestinal involvement, however, remains to be clarified (29, 34). Polymorphisms in the *IL10* gene, particularly rs1800896, a promoter variant associated with reduced IL-10 production, have been linked to increased IBD susceptibility across multiple populations (35). The reported frequency of this variant is relatively high in Turkish IBD cohorts but lower in East Asian cohorts, including Japan; this pattern reflects cohort-specific genetic variation rather than a simple Europe–Asia distinction. Given that IL-10 biology is more strongly linked to ulcerative colitis than Crohn’s disease, this distribution aligns with the markedly higher prevalence of UC in Japan and the UC-like intestinal phenotype observed in East Asian BD. Nevertheless, variation in IL-10 alone does not fully account for the East–West differences in intestinal BD, implying contributions from additional environmental and immunoregulatory factors. Beyond polygenic risk, loss-of-function mutations in *IL10*, *IL10RA*, and *IL10RB* cause severe monogenic forms of very early-onset IBD, underscoring the essential role of IL-10 signaling in maintaining mucosal immune equilibrium (36).

In BD, IL-10 is suggested to have a protective role, supported by evidence that monocytes from individuals homozygous for the A allele at *IL10* rs1518111 produce lower IL-10 levels following lipopolysaccharide stimulation. This SNP was strongly associated with BD in Turkish cohorts (p = 1.88 × 10^−8^) and shown to reduce IL10 mRNA expression and protein production (37). However, it did not reach genome-wide significance in Japanese cohorts (p = 5.89 × 10^−8^), despite the higher prevalence of intestinal BD in East Asia. While this may suggest a population-specific influence of IL-10 signaling, it also indicates that variation in IL-10 alone is insufficient to account for geographic differences in intestinal BD prevalence (37).

## A closer look at MEFV in both FMF and BD

**Figure 2:**
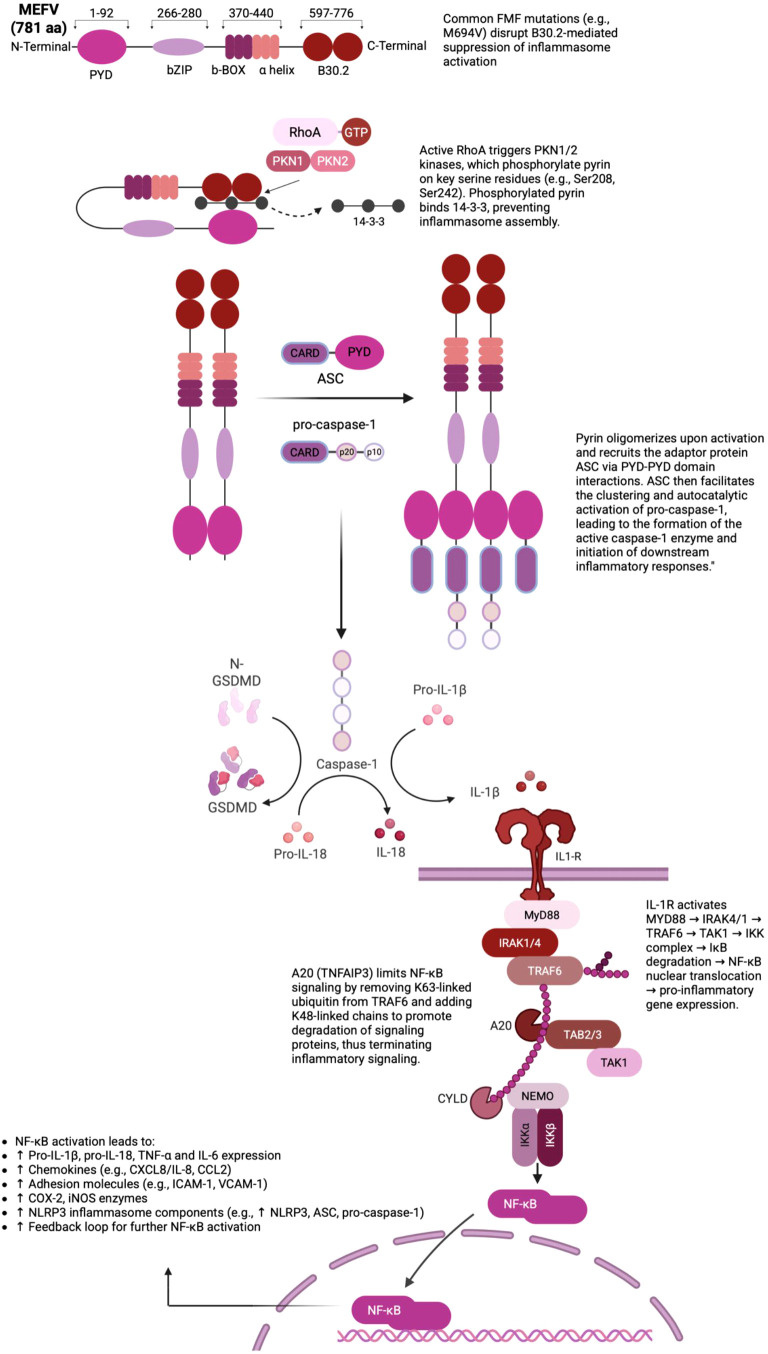
Schematic representation of pyrin inflammasome activation and downstream inflammatory signalling. Pyrin, under normal conditions, is kept inactive by phosphorylation of serine residues (e.g., Ser208 and Ser242) via RhoA-mediated activation of kinases such as PKN1/2. This phosphorylation enables 14-3-3 proteins to bind and inhibit pyrin. Upon RhoA inactivation or in the presence of MEFV gain-of-function mutations (e.g., M694V), pyrin is dephosphorylated, leading to dissociation from 14-3-3 and assembly of the pyrin inflammasome. This complex recruits ASC and procaspase-1, resulting in the activation of caspase-1, which cleaves pro-IL-1β and pro-IL-18 into their mature, secreted forms and induces pyroptosis via gasdermin D cleavage. In parallel, IL-1 receptor engagement triggers the MYD88-dependent pathway, culminating in NF-κB activation and transcription of pro-inflammatory genes. A20 (TNFAIP3) acts as a key negative regulator by deubiquitinating TRAF6 and limiting downstream NF-κB signaling, thus controlling inflammation.

One of the prototypic autoinflammatory or innate immunopathology-driven diseases is familial Mediterranean fever (FMF), caused by mutations in the pyrin-encoding *MEFV* gene (38). Gain-of-function mutations in pyrin, occurring in homozygous or compound heterozygous states or occasionally as a single mutated allele, are associated with FMF through activation of the pyrin inflammasome and subsequent IL-1β- and IL-18-driven inflammatory responses (38). The detailed molecular cascade of pyrin inflammasome activation and its downstream consequences is illustrated in **Figure 2**. FMF is common in the Eastern Mediterranean region (Turks, Jews, Arabs, and Armenians). Although hundreds of patients have been reported in Europe, the Americas, and Japan, these cases are largely attributed to immigration from endemic regions. The prevalence of FMF in endemic countries is between 1 in 500–1,000, with the highest prevalence reported as 1 in 395 in central Anatolia, followed by Israel and Armenia (38). Moreover, Yilmaz et al. found the *MEFV* carrier rate in the healthy Turkish population to be 20% (M694V 3%, M680I 5%, V726A 2%, M694I 0%, and E148Q 12%) (39). FMF is common in Turkey, the Middle East, Armenian, Arab, and Jewish populations, and its frequency decreases toward the east (40). Although comprehensive national registries are lacking, a hospital-based survey study from Japan estimated the number of FMF patients to be approximately 292, corresponding to a prevalence of about 1 in 435,000 (41). These findings suggest that FMF is exceptionally rare in the Japanese population.

While FMF is fundamentally a monogenic disease caused by *MEFV* mutations, clinical differences between populations suggest that additional layers of regulation may modulate disease expression. Epigenetic variation, environmental exposures, and microbiota composition have been proposed as potential modifiers of pyrin activity and downstream inflammation. The degree to which different *MEFV* mutations are functionally penetrant may also influence how susceptible a given population is to such modifying factors.

## MEFV mutations beyond FMF: their complex and context-dependent role in BD

The association between *MEFV* variants and BD remains uncertain. In Turkish cohorts, M694V has shown the most consistent signal, whereas other FMF-linked variants such as M680I and V726A have yielded inconsistent or null findings, especially in heterozygotes (42). In a Japanese cohort, no meaningful association between MEFV variants and BD was detected, underscoring potential population-specific effects and limited penetrance (42). Although the impact of *MEFV* carriage on the pathogenesis of BD is not fully understood, many studies have examined its relationship with distinct clinical manifestations of the disease. Atagündüz et al. (Turkey), Rabinovich et al. (Israel), and Seraslan et al. (Turkey) reported a higher frequency of vascular involvement in BD patients carrying *MEFV* mutations compared with non-carriers (43–45). However, Yazici et al. (Turkey) found vascular involvement less frequent among carriers, while Rabinovich et al. and Taşlıyurt et al. observed lower rates of uveitis and Seraslan et al. noted reduced arthritis frequency (44, 46, 47). In the study by Yazici et al., variants other than M694V were also included among *MEFV* carriers, and potential differences in disease duration, age, and sex distribution between carrier and non-carrier groups were not reported (46). In these studies, *MEFV* carriage was not associated with an increased frequency of intestinal BD, while vascular involvement appeared more common in *MEFV* carriers in most reports (48). Nevertheless, if any association exists, it appears largely confined to M694V, and this finding may still reflect population genetics rather than direct causality.

In a Japanese study, the relationship between intestinal BD and *MEFV* carriage was also investigated. *MEFV* gene analysis was performed in only 26 patients, a sample size too small to allow meaningful conclusions. Additionally, *MEFV* carriage was examined only in exon 2 and exon 3. *MEFV* mutation carrier rates were extremely high compared to population averages (healthy population: 38%—BD without intestinal involvement: 50%—intestinal BD: 75%) (49). *M694V* was detected very rarely in Japan in studies determining the frequency of *MEFV* carriage (50). This indicates that *M694V* has both potential detrimental and protective effects in the Japanese population as representative of the *MEFV* effect in BD. Overall, limited sample size and the lack of analysis of exon 10 mutations render the evidence insufficient to establish a definitive relationship (41).

## Neutrophil inflammasome activation and immunothrombosis via MEFV gain of function

MHC-I-opathy diseases such as spondyloarthritis (SpA), psoriasis, and BD are more common in FMF patients (51). One consistent explanation for this increase in frequency is that MHC-I-opathies have a strong neutrophil-driven innate immune system component. Moreover, the pathogenesis of MHC-I-opathies is based on the role of the IL-23/IL-17 axis in addition to class I-mediated peptide presentation (22). In FMF patients, preferential Th17 rather than Th1 polarization was demonstrated in *M694V* homozygous variants, especially at the time of attack (52). In addition, *ex vivo* stimulation of lymphocytes from FMF patients resulted in higher IL-17 and lower IFNγ production compared to healthy controls (53). In the context of MHC-I-opathy concept, type 17 inflammation provides increased neutrophil migration to the target site, and in FMF-MHC-I-opathy overlaps, pyrin may be one of the potential inducers of type 17 inflammation.

**Figure 3:**
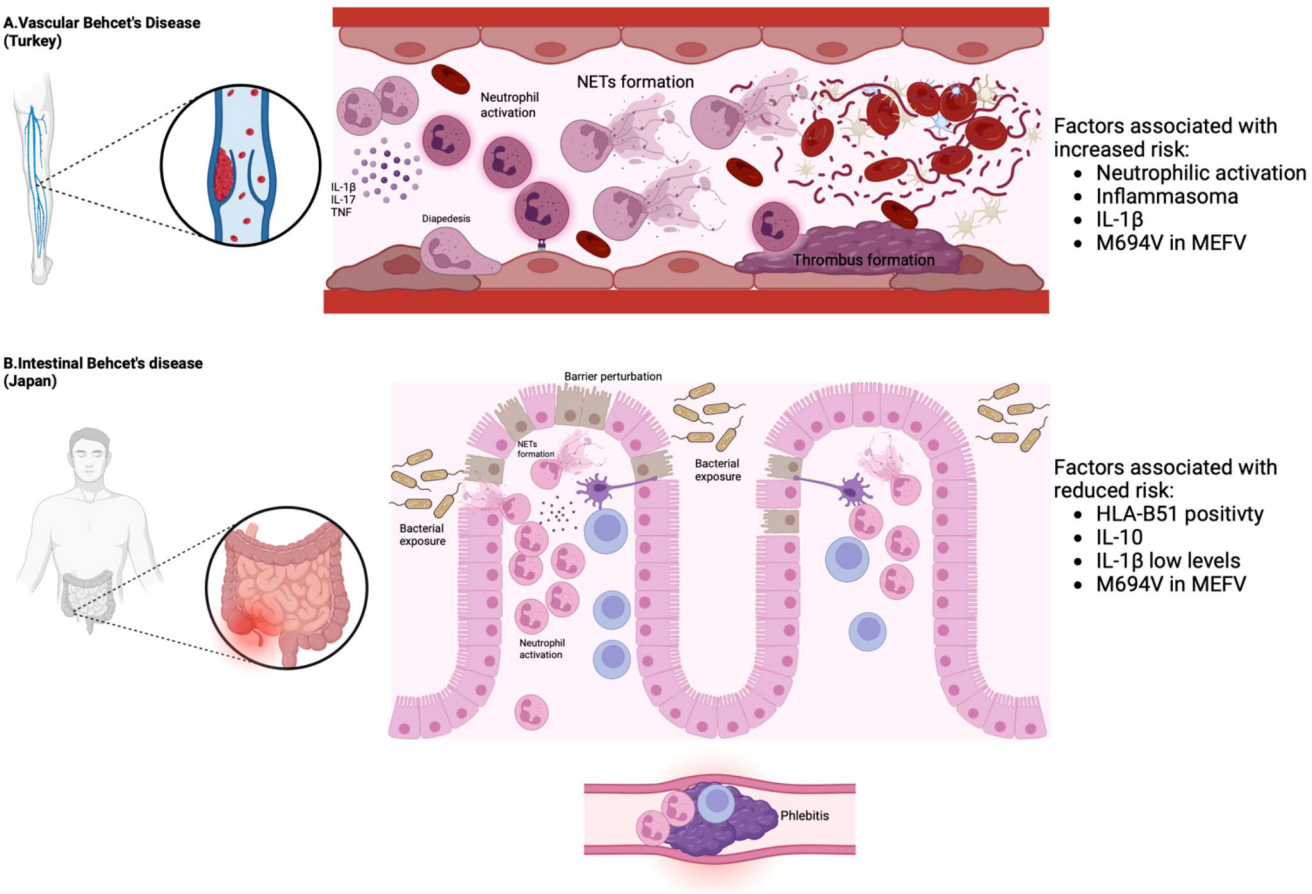
Factors contributing to vascular and intestinal inflammation in Behçet's Disease. Factors Contributing to Vascular and Intestinal Inflammation in Behçet's Disease. The underlying factors responsible for the differing prevalence of intestinal and vascular Behçet's disease (BD) across populations are most evident in Turkish and Japanese cohorts, where this disparity is most pronounced. A notable factor is the higher prevalence of HLA-B51 positivity in Turkey, which has been associated with a lower incidence of intestinal involvement in BD. However, HLA-B51 positivity alone is insufficient to fully account for this phenotypic divergence. Additionally, IL-10, a cytokine known to maintain intestinal barrier integrity, may contribute to an increased risk of intestinal inflammation, similar to its role in inflammatory bowel diseases. Furthermore, low-dose IL-1B has been shown to support intestinal barrier integrity. Given the high prevalence of MEFV mutations in Turkish BD populations, the elevated MEFV carriage in Turkey may represent a protective factor against intestinal inflammation in BD.

Thrombo-inflammation in vascular BD is thought to progress by the recruitment of stimulated neutrophils to sites of endothelial damage (**Figure 3**). By increasing the production of reactive oxygen species (ROS), triggering structural changes in fibrin, and extrusion of neutrophil extracellular traps, these processes effectuate the cardinal features of thrombo-inflammation in vascular BD (6). Similarly, the increased frequency of *MEFV* carriage in polyarteritis nodosa (PAN) is more than a coincidental association of this disorder with its neutrophil-driven nature (54). It is noteworthy that other vasculitis, such as IgA vasculitis, can develop on the basis of FMF as well as PAN (55). Additionally, the increased frequency of stroke in FMF patients in large population studies may also represent a predisposition to endothelial disorders (56). However, FMF itself is not characterized by true venous or arterial inflammation, in contrast to the vascular pathology observed in BD. Rather than directly causing vascular lesions, FMF may amplify endothelial or neutrophil activation in genetically or immunologically predisposed individuals, thereby enhancing disease-specific inflammatory responses and facilitating the emergence of certain vascular phenotypes. These associations are also consistent with a propensity for thrombosis in BD with *MEFV* carriage that is prevalent in Turkey but not Japan. IL-23R risk variants identified in Turkish BD cohorts may lead to enhanced IL-23 signaling, resulting in increased IL-17 production and neutrophil activation. This pro-inflammatory cascade promotes neutrophil-driven thrombo-inflammation, providing a mechanistic link to vascular pathology in BD (**Figure 3**).

Intriguingly, inflammasome activation via a different sensor (NLRP3) has been strongly linked to COVID-19-associated coagulopathy and immunothrombosis (57). IL-1β is a strong inducer for the release of tissue factor (TF)-rich microvesicles from macrophages (58), induces von Willebrand factor expression, and downregulates the expression of anticoagulant factors such as protein C and thrombomodulin (59, 60). Additionally, gasdermin D, a pore-forming protein activated upon cleavage by caspase-1 after inflammasome activation, may also contribute to thrombogenesis through facilitating NETosis in neutrophils (61) and permitting cellular calcium influx which in turn promotes TF procoagulant activity via phosphatidylserine externalization (62). IL-1α, released as an alarmin during cellular damage, promotes immunothrombosis by inducing endothelial activation, upregulating adhesion molecules, and enhancing tissue factor expression, thus amplifying thrombo-inflammatory cascades. Its role is distinct from IL-1β, acting locally as a membrane-bound cytokine that links tissue injury to thrombogenesis (63). IL-1 and IL-18 signaling is also well established as an important factor in arterial thrombosis (64). It is therefore unsurprising that *MEFV* carriage, and therefore increased inflammasome activity, is linked to increased thrombo-inflammation in BD (49). However, this mechanism does not translate into a high frequency of vascular events in FMF, most likely because the IL-1–mediated inflammation in FMF is typically episodic and serosal, resolving rapidly without sustained endothelial activation. In contrast, BD is characterized by chronic, low-grade vascular inflammation in which persistent cytokine release and neutrophil activation create ongoing endothelial stress that favors thrombosis. Thus, while *MEFV*-related inflammasome priming may enhance thrombo-inflammation in BD, it does not independently cause vasculitis in FMF (65).

Additionally, a meta-analysis of five studies conducted in Turkey investigated the association between IL-1 family gene polymorphisms and BD. The analysis revealed that the *IL1A*-889 C>T polymorphism was associated with a reduced risk of BD for both the CT and TT genotypes (28% and 39% lower, respectively), whereas the *IL1B*-511 C>T polymorphism (specifically the CC genotype and the C allele) was associated with an increased risk approximately 2-fold and 1.41-fold, respectively. Furthermore, the *IL1RN* (interleukin-1 receptor antagonist) intron 2 VNTR polymorphism, particularly the TT genotype of the *mspa1* allele (*IL1RN* 1100 TT), was associated with a 50% increased risk of BD, while the CT genotype appeared protective (66). Despite these associations, the precise functional impact of these polymorphisms on IL-1 activity and their mechanistic role in BD pathogenesis remain unclear since similar studies have not yet been conducted in East Asian populations, including Japan, highlighting the need for further research across diverse ethnic groups to validate and expand upon these findings (27).

## Insights from animal models

It has been shown that mice with IL-10 or IL-10 receptor deficiency through genetic intervention develop spontaneous colitis (67). Specifically, in a murine model, biopsy-induced colon injury increased macrophage-derived IL-10 at the site of injury and induced epithelial proliferation by stimulating the synthesis of Wnt1-inducible signaling protein-1 (68). In addition, the absence of T and B lymphocytes (i.e., the adaptive immune system) in these animal models did not impair the proliferative and healing effect in the wound area, indicating that macrophage-derived IL-10 was sufficient (68).

Notably, the most striking finding regarding the potential role of pyrin protein is the detection of more severe colitis in the colon of *MEFV* ^−/−^ mice compared to controls after stimulation with dextran sodium sulfate (DSS). In mice with DSS-induced colitis, pyrin promoted epithelial barrier integrity especially via IL-18, reducing epithelial permeability, intestinal inflammation, colitis severity, and even tumour formation (69). Indeed, several murine studies using genetic knockout models have shown that loss of key inflammasome components, including *Nlrp3*, *ASC*, C*aspase-1 (*70*)*, and *Nlrp1b (*71*)*, results in increased susceptibility to colitis, highlighting a protective role of inflammasome-mediated pathways in intestinal homeostasis. In these murine models, loss of inflammasome components leads to high susceptibility to DSS-induced colitis and severe DSS-induced intestinal inflammation. As a key regulator of the intestinal immune microenvironment, the inflammasome may play a pivotal role in modulating the pathogenesis of human IBD and intestinal BD.

While elevated levels of IL-1β and IL-18 are typically thought of as pathological cytokines in intestinal biology, they also have important protective roles in homeostasis. Both cytokines are involved in regulatory T-cell (Treg) maintenance, and notably, IL-18 (particularly in combination with IL-12) is essential for the activation of mucosal-associated invariant T (MAIT) cells, which contribute to mucosal immune defense (72). IL-1β induces IL-2 secretion from ILC3s, an important survival factor for Treg cells (73). IL-1β also induces CSF2 secretion from ILC3s, thereby promoting IL-10 and retinoic acid production from dendritic cells, both of which have anti-inflammatory effects and maintain Treg cell populations (74). In addition to the abovementioned data, IL-18 has also been shown to be vital for Treg-mediated control of intestinal inflammation in *IL-18R1^−−-^* mice where loss of IL-18 signaling leads to increases in colonic Th17 populations (75). Having BD on a *MEFV* background may therefore provide a level of intestinal protection through promoting Treg populations as a result of increased IL-1 and IL-18 signaling.

## Clues from therapy pointing to role of IL-1

**Figure 4:**
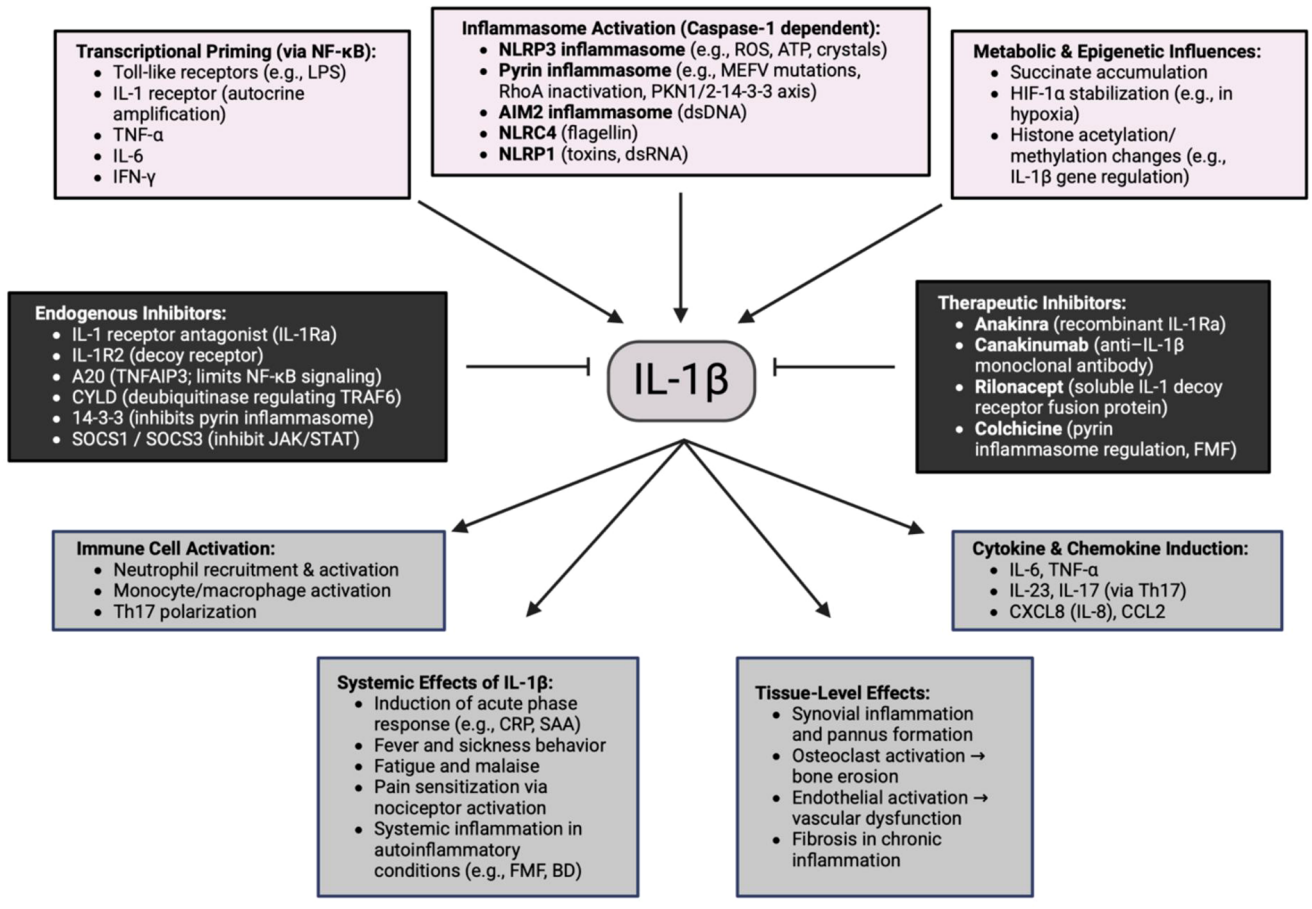
Regulatory inputs and downstream effects of IL-1β in inflammation. Regulatory inputs and downstream effects of IL-1β in inflammation. This schematic summarizes the major pathways influencing IL-1β production and its multifaceted biological effects. Upstream drivers include transcriptional priming via NF-κB, inflammasome activation (notably pyrin and NLRP3), metabolic/epigenetic modulation, and immune triggers. IL-1β acts on multiple levels: promoting immune cell activation, systemic inflammatory responses, cytokine and chemokine production, and tissue-level changes including bone erosion and fibrosis. Endogenous and therapeutic inhibitors of IL-1β are also depicted. The central role of pyrin, particularly in autoinflammatory conditions, is highlighted among inflammasome pathways.

An integrated overview of IL-1β regulation and its systemic and tissue-specific effects is provided in **Figure 4**, highlighting its dual role in inflammation and homeostasis. The complex immunopathogenesis of human BD cannot be fully recapitulated in animal models, which underscores the importance of human immunogenetics to decipher the different BD organ pathologies across different populations. It is worth pointing out that before transgenic animal technology with both knock-in and knock-out models for cytokine and immune system function, the “state of the art” for understanding cytokine function in experimental immune disease was either administering a cytokine or its neutralisation with monoclonal antibody therapy. Such cytokine blocking strategies were used to understand the role of cytokines in arthritis and demyelination including TNF, IL-1β, and IL-6. Likewise, in the modern era of cytokine pathway antagonism in man, it is possible to integrate immunogenetics and translational therapeutics to better decipher BD immunopathogenesis and the associated gut immunopathology (76).

As an example, blocking IL-17A, a cytokine downstream of IL-1 stimulation of Type 17 T cells, is ineffective in IBD (77). However, for BD-related enteritis, the limited evidence suggests the opposite is true, whereby IL-17A blockade is linked to good efficacy in the intestine (78). Mechanistically, how could this paradoxical scenario arise in humans? In experimental settings, the loss of IL-23R signaling in T cells is associated with γδ T-cell production of IL-17A that protects the intestinal barrier through maintenance of tight junctions (79). This is thought to be related to IL-1β whereby low levels associated with intestinal protection might be linked to MEFV heterozygous carriage, whereas higher levels could actually drive IL-1-mediated IL-17 inflammation (65, 80). It must be noted that there is a close relationship between IL-1 and IL-17 since IL-1 protects again septicemia in IL-17 KO mice. In the study conducted by Watad et al. in FMF patients, the high prevalence of IBD indicates the emergence of the IL-23-dependent or independent effect of increased IL-1β in a pathogenic context, rather than the intestinal protective effect of MEFV carriage (51).

A second example relates to the use of Janus kinase inhibitors (JAKi) in intestinal BD. Again, while the number of cases is small, the JAK inhibitor tofacitinib was generally effective in 15 BD but did not improve intestinal manifestations (81). However, there are also case reports where tofacitinib is effective in intestinal BD (82). Given the link between BD and IL-10 SNPs and the known protective role of IL-10 in the gut, we propose a mechanism of IL-10 loss of function under JAK as an explanation for this finding, as predicted by O’Shea and colleagues in the past (83).

## Conclusions

We have emphasized the potential impact of genetic factors that likely contribute to the ethnic and phenotypic diversity of Behçet’s disease (BD). In particular, we explored the possible roles of IL-1β-, IL-10-, and IL-23-driven mechanisms as key components of various pathogenetic processes in BD in relation to gut and vascular organ involvement. Additionally, the potential role of the *MEFV* gene, primarily acting through IL-1β, is further supported by epidemiological data and experimental observations linking it simultaneously to increased thrombosis risk and potential intestinal protection. This diversity in inflammatory response may also reflect broader immunogenetic differences across populations. While NOD2-driven pathways play a central role in Western cohorts with intestinal inflammation, East Asian populations appear to rely more on alternative immune mechanisms, such as TNF- or IL-17-related responses. In populations with high *MEFV* carriage, IL-1–mediated inflammation may also represent a compensatory or alternative axis. Given the role of NOD2 in microbial sensing, NF-κB activation, and IL-1β secretion, its absence in East Asians may indicate a shift toward distinct inflammatory circuits. These contrasts underscore the importance of tailored therapeutic strategies, as IL-23– or IL-1–targeted therapies may yield differing outcomes depending on underlying population-specific mechanisms.

In addition to immunogenetic factors, environmental contributors such as the intestinal microbiota may play a role in shaping BD phenotypes. Perturbation of barrier function and increased bacterial product exposure have been implicated in mucocutaneous inflammation and may similarly influence intestinal involvement. Dietary and microbiotal differences between Turkey and Japan remain underexplored, and future research should aim to stratify patients by relevant clinical and environmental features across ethnically diverse populations. A more refined classification of such variables may ultimately help delineate cytokine-driven organ-specific inflammation in BD and support more individualized therapeutic targeting. Interestingly, both vascular and intestinal BD manifestations show robust clinical response to TNF blockade (84, 85), whereas evidence supporting IL-1 inhibition in vascular BD remains limited and largely anecdotal. This discrepancy suggests that while IL-1–driven inflammasome activation may provide the initial inflammatory priming, sustained vascular and mucosal inflammation in BD is largely maintained through downstream cytokine circuits involving TNF, IL-23, and IL-17. Understanding how these pathways intersect across different disease phenotypes may help explain the variable therapeutic responses observed.
